# Progressive in vivo development of resistance to cefiderocol in *Pseudomonas aeruginosa*

**DOI:** 10.1007/s10096-022-04526-0

**Published:** 2022-11-15

**Authors:** Mustafa Sadek, Rémi Le Guern, Eric Kipnis, Philippe Gosset, Laurent Poirel, Rodrigue Dessein, Patrice Nordmann

**Affiliations:** 1 Medical and Molecular Microbiology, Faculty of Science and Medicine, University of Fribourg, Chemin du Musée 18, CH-1700 Fribourg, Switzerland; 2 Department of Food Hygiene and Control, Faculty of Veterinary Medicine, South Valley University, Qena, Egypt; 3 Center for Infection and Immunity of Lille, Université de Lille, CNRS, INSERM, CHU Lille, Institut Pasteur Lille, U1019-UMR 9017 Lille, France; 4 European Institute for Emerging Antibiotic Resistance, Pasteur Institute and University of Lille, France and University of Fribourg, Fribourg, Switzerland; 5 Swiss National Reference Center for Emerging Antibiotic Resistance (NARA), University of Fribourg, Fribourg, Switzerland; 6 Institute for Microbiology, University of Lausanne and University Hospital Centre, Lausanne, Switzerland

**Keywords:** Cefiderocol, *Pseudomonas aeruginosa*, Iron transporters, In vivo

## Abstract

We report in vivo development of cefiderocol (FDC) resistance among four sequential *Pseudomonas aeruginosa* clinical isolates ST244 recovered from a single patient, without exposure to FDC, which raises concern about the effectiveness of this novel drug. The first recovered *P. aeruginosa* isolate (P-01) was susceptible to FDC (2 μg/mL), albeit this MIC value was higher than that of a wild-type *P. aeruginosa* (0.12–0.25 μg/ml). The subsequent isolated strains (P-02, P-03, P-04) displayed increasing levels of FDC MICs (8, 16, and 64 μg/ml, respectively). Those isolates also showed variable and gradual increasing levels of resistance to most β-lactams tested in this study. Surprisingly, no acquired β-lactamase was identified in any of those isolates. Whole-genome sequence analysis suggested that this resistance was driven by multifactorial mechanisms including mutational changes in iron transporter proteins associated with FDC uptake, *ampC* gene overproduction, and *mexAB-oprM* overexpression. These findings highlight that a susceptibility testing to FDC must be performed prior to any prescription.

*Pseudomonas* *aeruginosa* is one of the most frequent nosocomial pathogens, particularly as a source of acquired pneumonia in intensive care units with a tendency towards multidrug resistance [1]. Cefiderocol (FDC) is a novel siderophore cephalosporin that shows activity against most multidrug-resistant *P. aeruginosa* strains, including carbapenem-resistant *P. aeruginosa* [2]. Its broad and excellent activity is explained by its unique and so-called Trojan horse strategy relying on the active penetration into Gram-negative bacterial cells using its iron transport system [3]. There is some evidence of acquired resistance to FDC. Nevertheless, it seems that the potential for resistance acquisition remains low [4, 5]. The mechanisms underlying this resistance remain poorly understood. Resistance to FDC in *P. aeruginosa* has been demonstrated to be associated with alterations of iron uptake pathways [6], structural modification of the natural AmpC β-lactamase [7], and modification of the expression of efflux systems. The aim of this study was to decipher the mechanisms and associated genetic determinants responsible for increased resistance pattern to FDC among four sequential *P. aeruginosa* isolates recovered from a single patient who had never been treated with FDC.

## Case history

A 65-year-old patient was hospitalized in the surgical intensive care unit of the Lille University Hospital for acute respiratory distress syndrome following pancreaticoduodenectomy. His medical history included fibrosing interstitial lung disease and rheumatoid arthritis. This patient developed ventilator-acquired pneumonia, for which four sequential clinical isolates of *P. aeruginosa* (P-01, P-02, P-03, and P-04) were recovered over a period of 3 weeks. The first and second isolates (P-01 and P-02) were from tracheal aspirate samples during the first week of ventilator-associated pneumonia. The second isolate (P-02) was already resistant to all tested β-lactams except ceftolozane-tazobactam (C/T). The patient was treated with meropenem (1 g q8h) IV associated with colistin IV (9 MUI q12h) because C/T was unavailable due to long-term drug shortage. The third and fourth isolates (P-03 and P-04) were recovered from tracheal aspirate samples during the second and third week of treatment, respectively.

## Methods

Antimicrobial susceptibility testing was performed by using the disk diffusion method on Mueller–Hinton agar plates for selected antibiotics. Minimum inhibitory concentrations (MICs) were then determined using Etest strips (bioMérieux, La Balme-les-Grottes, France) on Mueller–Hinton agar plates at 37 °C for all antibiotics or antibiotic combinations except for FDC and colistin. MIC values of FDC were determined with the reference BMD method using iron-depleted cation-adjusted Mueller–Hinton (ID-CAMH) broth prepared following the protocol described by Hackel et al. [8]. MICs of colistin were determined using broth microdilution in cation-adjusted Mueller–Hinton broth (Bio-Rad). The results were interpreted according to the latest EUCAST breakpoints (https://www.eucast.org/fileadmin/src/media/PDFs/EUCAST_files/Breakpoint_tables/v_12.0_Breakpoint_Tables.pdf) [9]. The reference strain *P. aeruginosa* ATCC 27,853 was used as quality control for all testing.

Whole-genome sequencing (WGS) was performed for the four isolates with the ultimate goal to investigate the molecular mechanisms underlying such resistance pattern. The entire genome was sequenced using a MiSeq Illumina platform (Illumina, San Diego, CA, USA) using the Nextera sample preparation method with 2 × 150 bp paired end reads. Illumina short reads were assembled using the CLC Genomic Workbench (version 20.0.4; CLC Bio, Aarhus, Denmark), and contigs with a minimum contig length of 800 nucleotides (nt) were generated. The resulting assembled sequences were analyzed using ResFinder 4.1 software (for antimicrobial resistance genes) and MLST 2.0 software (for Multilocus sequence typing (MLST) analysis on the Center for Genomic Epidemiology server (http://www.genomicepidemiology.org/). The raw sequence data project had been deposited at GenBank under accession no PRJNA856996.

## Results

According to results of antimicrobial susceptibility testing and considering the EUCAST resistance breakpoint for FDC at > 2 μg/ml, the first *P. aeruginosa* isolate recovered (P-01) was susceptible to FDC (2 μg/ml), albeit this MIC value was higher than that of wild-type *P. aeruginosa* (0.125–0.25 μg/ml). The subsequent isolates (P-02, P-03, P-04) displayed gradual elevation of FDC MICs (8, 16, and 64 μg/ml, respectively) (Table 1). Those *P. aeruginosa* isolates showed also variable and increasing levels of resistance to most β-lactams including meropenem (8 to > 32 μg/ml), meropenem/vaborbactam (4 to > 64 μg/ml), imipenem (> 32 μg/ml), imipenem/relebactam (3 to > 32 μg/ml), ceftazidime (16 to > 256 μg/ml), ceftazidime/avibactam (1.5 to > 256 μg/ml), ceftolozane/tazobactam (0.75 to > 2 μg/ml), cefepime (8 to 48 μg/ml), aztreonam (8 to > 256 μg/ml), tetracycline (2 to > 256 μg/ml), and chloramphenicol (> 256 μg/ml). The MIC of colistin remained unchanged and was found to be 1–2 μg/ml for all those isolates.

**Table 1:** MIC determination and genomic features of *P. aeruginosa* isolates with progressive in vivo development of resistance to cefiderocol

Isolate	FDC3850S (P-01)	FDC3850R (P-02)	C0197 (P-03)	C4728 (P-04)
**ST**	244	244	244	244
**MIC (mg/L)**				
FDC	2	8	16	64
FDC + cloxa 2000	0.5	2	2	2
IPM	> 32	> 32	> 32	> 32
I-R	3	4	12	> 32
MEM	8–16	> 32	> 32	> 32
MVB	4	32	> 64	> 64
CAZ	16	64	> 256	> 256
CZA	1.5	24	24	> 256
FEP	8	16	32	48
ATM	8	64	128	> 256
TET	12	> 256	> 256	> 256
CHL	> 256	> 256	> 256	> 256
**β-lactamases**	PDC-1, OXA-847	PDC-1, OXA-847	PDC-1, OXA-847	PDC-1, OXA-847
**Other resistance genes**	*catB7*, *aph(3')-IIb*, *dfrA43*, *fosA*, *carA*[2], *arnA*	*catB7*, *aph(3')-IIb*, *dfrA43*, *fosA*, *carA*[2], *arnA*	*catB7*, *aph(3')-IIb*, *dfrA43*, *fosA*, *carA*[2], *arnA*	*catB7*, *aph(3')-IIb*, *dfrA43*, *fosA*, *carA*[2], *arnA*
**TonB-dependent receptor proteins**				
PirS	–	–	–	–
PiR	–	–	–	–
PirA	A370T	A370T	A370T	A370T
PiuA	Q38H	Q38H	Q38H	Q38H
PiuB	I343L, AN573-574TD	I343L, AN573-574TD	I343L, AN573-574TD	I343L, AN573-574TD
PvdS	–	–	–	–
ExbB	–	–	–	–
ExbD	–	–	–	–
TonB3	F35L, V122A	F35L, V122A	F35L, V122A	F35L, V122A
**Other proteins of interest**				
NalC	–	–	–	–
NalD	–	–	–	–
MexR	–	D89E	D89E	D89E
MexA	–	–	–	–
MexB	–	–	–	V767G
OprM	–	–	–	–
NfxB	–	–	–	–
MexC	R76Q, H309R, S330A, P383S	R76Q, H309R, S330A, P383S	R76Q, H309R, S330A, P383S	R76Q, H309R, S330A, P383S
MexD	S845R	S845R	S845R	S845R
OprJ	DM68-69GV; 12 aa changes at positions 314–344	DM68-69GV	DM68-69GV	DM68-69GV
ParR	–	–	–	–
ParS	H398R	H398R	H398R	H398R
MexS	A75V, G244D, D249N	A75V, G244D, D249N	A75V, G244D, D249N	A75V, G244D, D249N
MexT	Truncated at aa 80	Truncated at aa 80	Truncated at aa 80	Truncated at aa 80
MexE	–	–	Truncated at aa 40 (frameshift mutation starts at AA40 and stop codon at AA82)	Truncated at aa 58 (Del four AAs (58–61) and truncated at AA82 via stop codon)
MexF	–	–	–	–
OprN	–	–	–	–
MexL	–	–	–	–
MexJ	–	–	–	–
MexK	–	–	–	–
OprD	Truncated at aa 90 (frameshift mutation)	Truncated at aa 70 (frameshift mutation)	Truncated at aa 70 (frameshift mutation)	Truncated at aa 70 (frameshift mutation)
AmpC	PDC-1	PDC-1	PDC-1	PDC-1
AmpE	ND	ND	ND	ND
AmpD	ND	ND	ND	ND
AmpDh2	–	–	–	–
AmpDh3	A219T	A219T	A219T	A219T
AmpR	–	–	–	–
FecA	T339A, H363R	T339A, H363R	T339A, H363R	T339A, H363R
FiuA	M670I, A677T, T771A	M670I, A677T, T771A	M670I, A677T, T771A	M670I, A677T, T771A
FiuI	–	–	–	–
FptA	–	–	–	–
FpvA	–	–	–	–
FpvB	T67K, A125T, E262D	T67K, A125T, E262D	T67K, A125T, E262D	T67K, A125T, E262D
PBP1A	Ins 615P616	Ins 615P616	Ins 615P616	Ins 615P616
PBP1B	S96N	S96N	S96N	S96N
PBP2	–	–	–	–
PBP3	–	–	–	–
PBP3A	–	–	–	–

*ST* sequence type, *MIC* minimum inhibitory concentration, *FDC* Cefiderocol, *Cloxa* cloxacillin, *IPM* imipenem, *I-R* imipenem/relebactam, *MEM* meropenem, *MVB* meropenem-vaborbactam, *CAZ* ceftazidime, *CZA* ceftazidime-avibactam, *FEP* cefepime, *ATM* aztreonam, TET tetracycline, *CHL* chloramphenicol, *CIP* ciprofloxacin, – no mutation, *ND* not detected, *PBP* penicillin binding protein

All isolates possessed identical intrinsic β-lactamase genes (*bla*_OXA-847_ [encoding a narrow-spectrum oxacillinase], *bla*_PDC-1_ [encoding an AmpC-type cephalosporinase]) but no acquired β-lactamase were identified. All four *P. aeruginosa* strains were found to belong to sequence-type ST244, one of the most widely distributed clones worldwide. WGS data of the four strains were analyzed compared to the reference strain *P. aeruginosa* PAO1 (GenBank no. AE004091.2).

Considering that no acquired β-lactamases were found in any of those isolates, efforts were directed towards examining *P. aeruginosa* targets potentially involved in other known resistance to FDC. These included insertions, deletions, and mutations in *pirS*, *piR*, *pirA*, *piuA*, *piuB*, *exbB-exbD-tonB3* or mutations in the *pvdS*, *fecI*, *fecR*, *fecA*, *fpvA*, *fpvB*, and *fiuA*—all components of the bacterial iron transport system. Also, proteins associated with impaired permeability were also assessed [7, 12]. Several identical mutations were identified in various TonB-dependent receptor proteins in all four strains including *pirA* (A370T), *piuA* (Q38H), *piuB* (I343L, AN573-574TD), *TonB3* (F35L, V122A), *fvpB* (T67K, A125T, E262D), *fecA* (T339A, H363R) and *fiuA* (M670I, A677T, T771A) (Table 1). A single amino acid substitution (D89E) was identified in MexR, the negative regulator of the MexAB-OprM efflux pump, in the three FDC-resistant strains (P-02, P-03, P-04) but not in the FDC-susceptible strain P-01.

To evaluate whether this reduced susceptibility to FDC could be related to increased expression of the *ampC* gene, MIC values for FDC were determined in combination with cloxacillin, an inhibitor of AmpC activity (Table 1). Hence, MICs of FDC dropped to 0.5, 2, 2, and 2 μg/ml for P-01, P-02, P-03, and P-04, respectively, when combined with cloxacillin at a fixed concentration of 2000 μg/ml. Further analysis of the WGS data identified the absence of the *ampD* gene in all isolates, this feature being known to induce upregulation of the *ampC gene*. Moreover, a single amino acid substitution (Ala219Thr) was identified in AmpDh3 of the four isolates analyzed. By analyzing the outer membrane proteins of *P. aeruginosa*, the OprD outer membrane porin, which is involved in carbapenem uptake, was found to be truncated in all four strains that were actually resistant to carbapenem.

## Discussion

The siderophore cephalosporin cefiderocol is one of the most promising commercialized agents against carbapenem-resistant Gram negatives including carbapenem-resistant *P. aeruginosa*. Although still uncommon, reduced susceptibility or resistance to FDC is being reported in that species. In this study, four sequential *P. aeruginosa* isolates were recovered from a single patient and analyzed using WGS. These isolates showed an increasing trend of resistance to broad-spectrum spectrum cephalosporins, carbapenems, and FDC over the time, without history of FDC exposure. Using the complete genome sequence data of the four *P. aeruginosa* isolates, all strains were shown to belong to the same sequence type, namely, ST244, a globally disseminated high-risk clone [10, 11].

The same mutations were detected in various iron transporters (*pirA*, *piuA*, *piuB*, *TonB3*, *fvpB*, *fecA* and *fiuA*), responsible for the active transport of FDC, for the four strains. These mutations may play an essential role in the basic increase of FDC MICs in all four strains and could be a potential mechanism of FDC resistance development in *P. aeruginosa* as previously reported [13–16]. However, those mutations in corresponding genes cannot explain by themselves the difference of susceptibility to FDC between those four strains.

The amino acid substitution found in MexR in the three FDC-resistant strains (P-02, P-03, P-04) was likely resulting in the overexpression of the *mexAB-oprM* multidrug efflux pump genes that may contribute to additional FDC resistance in those three strains as previously reported [17]. No mutation in the genes encoding TonB-dependent receptors (TBDRs) was identified in those isolates [17]. Very recently, Ikawa et al. indicated that the MexAB-OprM drug efflux system contributes to the intrinsic resistance to FDC in *P. aeruginosa* and found that the *mexAB-oprM*-deficient *P. aeruginosa* mutant displayed increased FDC susceptibility compared to the wild-type strain. On the other hand, the overexpression of *mexAB-oprM* in the *mexAB-oprM*-deficient mutant increased the MIC value of FDC [18]. In another study, MIC of FDC was slightly affected by the *oprD* gene deficiency or the overproduction of MexAB-OprM multidrug efflux pump due to a defect in the *mexR* or *nalD* regulatory genes in *P. aeruginosa* [4]. Although the association between MexAB-OprM overproduction and reduced FDC activity is still poorly understood, the role of *mexAB-OprM* overexpression in reducing FDC activity needs to be further explored as FDC could be a substrate of this efflux pump. Interestingly, truncation of the OprD-encoding gene had been previously reported among in vivo selected FDC-resistant clinical *P. aeruginosa* isolates (after FDC treatment) [14].

MICs of FDC were significantly decreased in the presence of cloxacillin, suggesting that overexpression of *ampC* gene was involved in the increased MICs of FDC in those four *P. aeruginosa* isolates. This increased expression of the *ampC* gene could be explained by the inactivation of *ampD gene* in the four isolates as previously described [19–22]. In a study, mutations in the *ampD* gene were associated with modest increases in FDC MICs to 2 μg/ml, remaining in the susceptible range in that latter case [17]. Moreover, the amino-acid substitution (Ala219Thr) identified in AmpDh3 of the four isolates might play an important role in overproduction of cephalosporinase leading to ceftazidime resistance, as previously reported [23], which likely cause collateral resistance to FDC since FDC combines chemical moieties of ceftazidime and cefepime. We noticed that the FDC-resistant *P. aeruginosa* strains (P-02, P-03, and P-04) showed a 4- to tenfold increase in FDC MICs compared to the FDC susceptible isolate (P-01). This increase in FDC MICs could be attributed to strong collateral-resistance with ceftazidime for which MICs were elevated by 4- to ≥ eightfold in those FDC-resistant *P. aeruginosa* strains compared to the FDC-susceptible isolate (P-01). Similar results were observed for cefepime and ceftazidime/avibactam (Table 1). Very recently, it has been shown that development of FDC resistance was often associated with collateral susceptibility changes towards other β-lactams such as increased resistance to ceftazidime and ceftazidime/avibactam [24].

In conclusion, we report here in vivo development of FDC resistance in *P. aeruginosa* clinical isolates without exposure to FDC is raising concerns about the effectiveness of this promising drug. Whole-genome sequence analysis suggests that this resistance was driven by multifactorial mechanisms including changes in iron transporter proteins, *ampC* gene overproduction, and *mexAB-oprM* overexpression.
